# COP9 Limits Dendritic Branching via Cullin3-Dependent Degradation of the Actin-Crosslinking BTB-Domain Protein Kelch

**DOI:** 10.1371/journal.pone.0007598

**Published:** 2009-10-27

**Authors:** Inna Djagaeva, Sergey Doronkin

**Affiliations:** Department of Anatomy and Neurobiology, University of Tennessee Health Science Center, Memphis, Tennessee, United States of America; Katholieke Universiteit Leuven, Belgium

## Abstract

Components of the COP9 signalosome (CSN), a key member of the conserved 26S proteasome degradation pathway, have been detected to be altered in patients of several debilitating syndromes. These findings suggest that CSN acts in neural circuits, but the exact function of CSN in brain remains unidentified. Previously, using *Drosophila* peripheral nervous system (PNS) as a model system, we determined that CSN is a critical regulator of dendritic morphogenesis. We found that defects in CSN led to the strikingly contrast phenotype of either reducing or stimulating dendritic branching. In particular, we have reported that CSN stimulates dendritic branching via Cullin1-mediated proteolysis. Here we describe that CSN inhibits dendritic arborization in PNS neurons acting via control of Cullin3 function: loss of Cullin3 causes excessive dendritic branching. We also identified a downstream target for Cullin3-dependent degradation in neurons – the actin-crosslinking BTB-domain protein Kelch. Inappropriate accumulation of Kelch, either due to the impaired Cullin3-dependent turnover, or ectopic expression of Kelch, leads to uncontrolled dendritic branching. These findings indicate that the CSN pathway modulates neuronal network in a multilayer manner, providing the foundation for new insight into the CSN role in human mental retardation disorders and neurodegenerative disease.

## Introduction

The morphological changes in dendritic development are essential for the proper wiring of the brain. One the most distinctive features of dendrites is their characteristic and extremely complex branching patterns. Dendritic shafts constantly extend and retract interstitial protrusions, or filopodia. During early development, some of these filopodia are stabilized into new dendritic branches, whereas later in development these dynamic filopodial extensions can develop into dendritic spines [1], [2].

Increasing evidence indicates that deficient structural neuronal network connectivity is a major, if not primary, cause of mental retardation [3]. A large number of disorders of the central nervous system are associated with altered dendritic spine numbers and morphology. These include multiple mental retardation disorders and autism spectrum disorders [4], [5], [6], [7], [8]. Aberrant spine morphology also occurs in psychiatric disorders [9] and drug addiction [10], as well as in neurodegenerative diseases including Alzheimer's disease [11], Huntington's disease [12], and Parkinson's disease [13]. Moreover, changes in the structure and function of dendritic spines contribute to numerous physiological processes such as synaptic transmission and plasticity, as well as behavior including learning and memory [14], [15], [16].

Dendritic morphogenesis and plasticity are based on rapid, dynamic remodeling of the actin cytoskeleton [4], [17], [18]. Proper actin changes are crucial for dendritic growth, guidance, and branching. Failures in actin rearrangements are associated with defects in neuronal wiring in the brain. Several genes that encode factors involved in actin cytoskeletal dynamics are mutated in individuals with mental retardation or autism spectrum disorders [7].

Because altered dendritic spine morphogenesis and plasticity are an endophenotype of many neurodevelopmental and neuropsychiatric disorders, the molecular mechanisms that control spine plasticity and pathology have been under intense investigation over the past few years. Understanding these mechanisms may provide clues as to how neurons become dysfunctional with age or disease.

Proteolysis is a major event in cellular metabolism and is commonly involved in a variety of regulatory pathways [19]. Rapid and substrate-specific protein degradation is among the most promising molecular switches to regulate actin remodeling during dendritic development [20], [21], [22]. A tight coordination between protein synthesis and degradation is critical for rapid response to the changing environment and, therefore, is expected in dendritic changes. However, despite growing progress in understanding molecular composition of the ubiquitin proteasome system, there are limited data about physiological aspects of this system, dendrites in particular.

One of the central components of the proteasome system is ubiquitin ligases [19]. They recognize proteins that have accomplished their duties and target them for degradation via proteasome. The Cullin-RING family of ubiquitin ligases shares conserved scaffold proteins of the Cullin family [23], [24]. There are five different Cullins: Cullin1 to Cullin5. The COP9 signalosome (CSN), a conserved eight-protein complex, is a major regulator of the Cullin-RING-based ubiquitin ligases [25], [26], [27]. CSN controls Cullin-based ubiquitin ligases by removing the Nedd8 modification from the Cullin component of the ligase [28], [29], [30], [31].

Compromised function of the CSN pathway has been detected in several mental retardation syndromes and neurodegenerative diseases such as Smith-Magenis syndrome, Down syndrome, Alzheimer's disease and Parkinson's disease, Machado-Joseph disease, X-linked mental retardation syndrome [32], [33], [34], [35], [36], [37], [38], [39], [40], [41]. However, the functional and mechanistic implications of CSN in neuronal development remain elusive. Using powerful *Drosophila* genetics and simple and convenient tools to analyze the peripheral nervous system in *Drosophila* larvae, we have been investigating the function of the CSN-mediated ubiquitin-dependent proteolysis in dendritic development. We have shown that CSN is critical for dendritic morphology (63). Loss of CSN function leads to a highly complex phenotype in dendrites, underlining the pleiotropic nature of CSN regulation. In particular, we detected both inhibiting and stimulating effects of CSN on dendritic elaboration. The stimulating effect of CSN on dendritic branching was translated via regulation of Cullin1-based ubiquitin ligase: loss of Cullin1 led to decreased dendritic branching (63). Given that CSN exerts its function via different Cullins, these findings suggest that CSN acts via other member(s) of the Cullin family to inhibit dendritic arborization.

Here we found that, in addition to Cullin1-mediated regulation, CSN controls dendritic morphogenesis via Cullin3. We found that Cullin3, in contrast to Cullin1, acts to prevent dendrites from excessive branching. We have identified the actin-crosslinking BTB domain protein Kelch as a downstream target of Cullin3-mediated proteolysis in the peripheral nervous system (PNS) neurons. Stabilization of Kelch in neurons either by mutations in *cullin3*, ectopic expression of Kelch, or both resulted in excessive dendritic branches and their overgrowth. These data provide a direct regulatory link between ubiquitin-dependent protein degradation, actin cytoskeleton, and neurodevelopment. Taken together, our results have exposed the dualistic role of the CSN signalosome in regulating the proper balance in dendritic arborization.

## Results

### Loss of *cullin3* stimulates neuronal development

**Figure 1:**
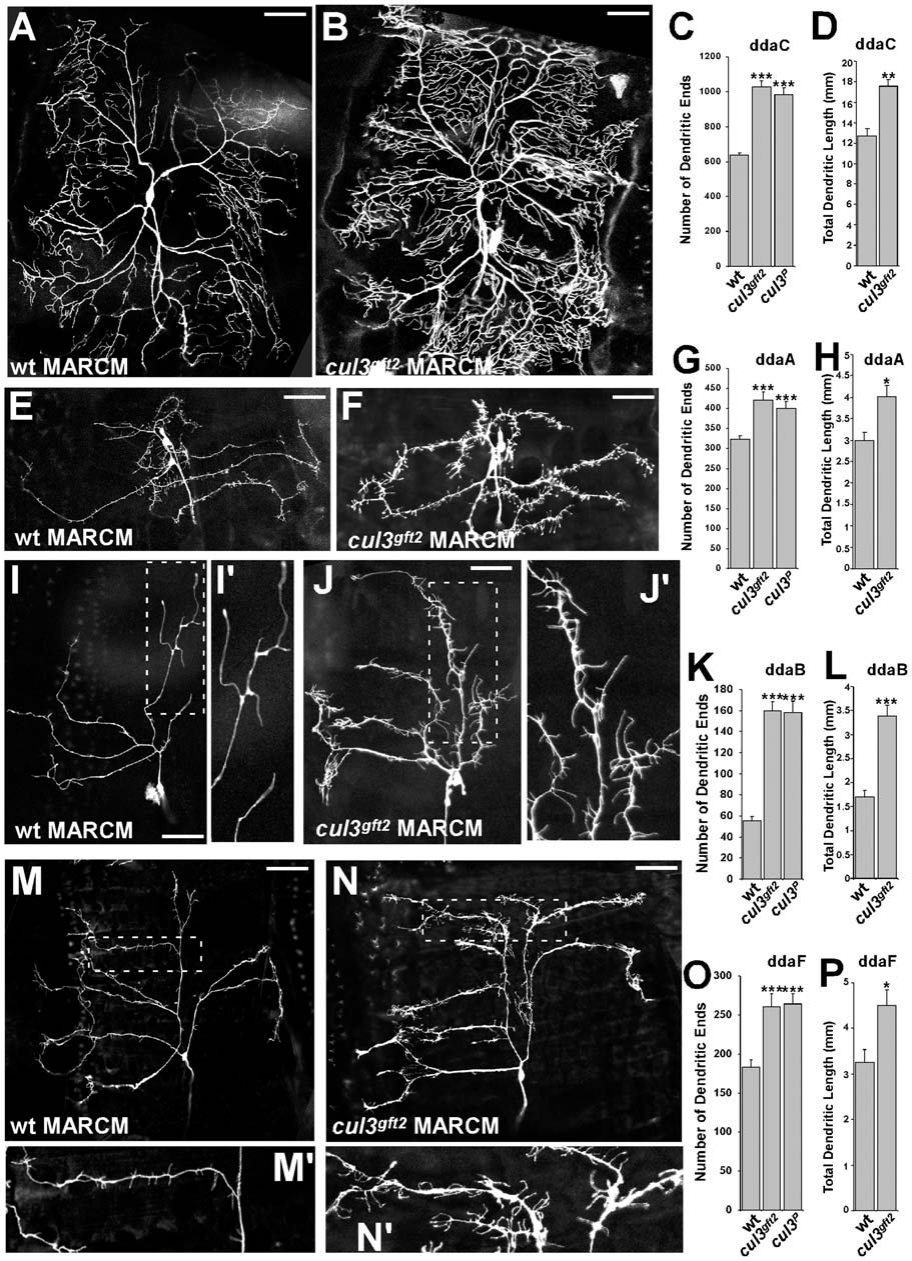
Loss of *cullin3* stimulates dendritic elaboration. (A, E, I, M) MARCM clones of wild type ddaC, ddaA, ddaB, ddaF neurons, respectively. (I′) and (M′) are magnified images from panels (I) and (M). In contrast to wild type, *cul3*-mutant ddaC (B), ddA (F), ddaB (J), ddaF (N) neurons show increasing dendritic branching. (J′) and (N′) are magnified images from panels (J) and (N). Scale bar: 50 µm. (C, G, K, O) Quantifications of terminal dendritic ends in wild type and two independent *cul3* alleles mutant ddaC, ddaA, ddaB, ddaF neurons, respectively. (D, H, L, P) Quantifications of total dendritic length in wild type and *cul3^gft2^* mutant ddaC, ddaA, ddaB, ddaF neurons, respectively. ***: p<0.001, **: p<0.01, *: p<0.02.

Previously we identified that CSN normally promotes dendritic branching via control of Cullin1 function, and prevents excessive dendritic branching through regulation of the Cullin3-dependent pathways (63). To investigate in details the Cullin3-mediated aspect of this regulation, we used *Drosophila* larval PNS neurons. Larval PNS neurons at the third instar stage elaborate characteristic, highly branched subepidermal patterns that can be visualized in living embryos or larvae with green fluorescent protein (GFP) [42] (Figure 1).

We used two strong independent alleles of *cul3: cul3^gft2^ and cul3^06430^*. Both alleles were lethal in first and second instar larvae, before larval PNS is fully developed. Therefore, we applied the MARCM technique [43], [44] to generate GFP-labeled *cul3*-homozygous clones in otherwise heterozygous and, therefore, viable larvae. Using this approach, we detected that *cullin3*-mutant neurons developed an excessively elaborated dendritic pattern (Figure 1B, F, J, N). Similar effects were observed in both independent *cul3* mutant alleles. We counted the number of dendritic ends of the third instar larval neurons in wild type and *cul3*-mutants (Figure 1C, G, K, O). Typically more branched ddaB, ddaC, and ddaF neurons were more elaborated in *cul3* mutant clones as well (Figure 1). In addition to the excessive branching, we detected increasing of the total dendritic length in *cul3* mutant clones (Figure 1D, H, L, P). We also noticed slightly increased dendritic field of *cul3-*mutant neurons, probably resulting from extensive branching and overgrowth (Table 1). Despite the excessive branching, general morphology of ddaB, ddaC, and ddaF *cul3*-mutant neurons was not affected, and every neuron was easily recognized.

**Table 1:** Quantification of total dendritic area of ddaC neurons.

Genotype	Average dendritic area (μm^2^), ×10^4^
wt	14.99+/−0.29
*cul3[gft2]*	15.85+/−0.56
*kel[DE1]*	12.42+/−0.44 (p<0.001)
*UAS-kel*	15.58+/−0.63
*cul3;UAS-kel*	19.23+/−0.65 (p<0.002)

**Figure 2:**
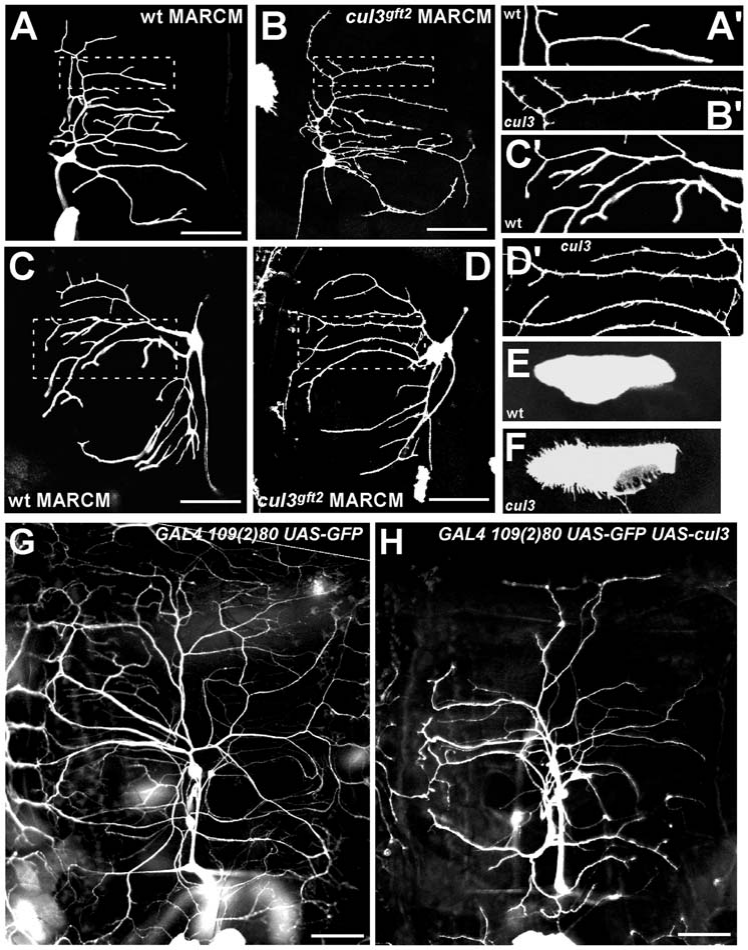
*cul3*-mutant neurons show small protrusions; Cullin3 overexpression represses dendritic development. (A, C) Typical wild type ddaE and ddaD neurons. (A′) and (C′) are magnified images from panels (A) and (C). (B, D) *cul3*-mutant ddaE and ddaD neurons. (B′) and (D′) are magnified images from panels (B) and (D). In *cul3* mutants, normally smooth ddaE and ddaD neurons generate small protrusions. (E) A wild type epithelial cell. (F) *cul3*-mutant epithelial cells with multiple protrusions. (G) PNS neurons in a non-mutant third instar larva, visualized by the *109(2)80-GAL4*-driven expression of GFP. (H) Overexpression of Cullin3 leads to repression of dendritic branching.

Strikingly, we detected that the appearance of the normally less branched neurons, ddaE and ddaD, was dramatically altered by *cullin3* mutations. In particular, we observed that loss of *cul3* generated small protrusions in ddaE and ddaD neurons (Figure 2B, D). In wild type, ddaE and ddaD neurons are smooth and do not generate protrusions (Figure 2A, C). In addition, *cul3*-mutant epithelial cells exhibited abnormal protrusions, when compared to the wild-type epithelial cells (Figure 2E, F).

Taken together, these findings demonstrated that *cullin3* is involved in dendritic branching.

### Elevated levels of Cullin3 inhibit dendritic branching

We examined whether the excess of Cullin3 affects dendritic branching. To do this, we generated a transgenic fly line that carries *pUAST-Cullin3* construct that produces the full-length Cullin3 protein. Ectopic expression of Cullin3 in PNS neurons was driven by the driver *109(2)80-GAL4* and visualized by GFP.

We found that Cullin3 overexpression led to severe reduction of the dendritic arborization (Figure 2H, vs 2G control). Thus, Cullin3 acts in PNS to restrict dendritic branching and keeps neurons from uncontrollable over-branching.

### Loss of *cullin3* causes accumulation of actin-crosslinking BTB domain protein Kelch

Because Cullin3 is a component of the ubiquitin-mediated protein degradation pathway, we wanted to detect its target(s) in neurons. It has been reported that Cullin3-based ligases bridge their targets by using BTB-domain-containing proteins [45], [46], [47], [48]. In addition, some BTB proteins can be degraded through the Cullin3 complex by an autocatalytic mechanism [45], [48].

**Figure 3:**
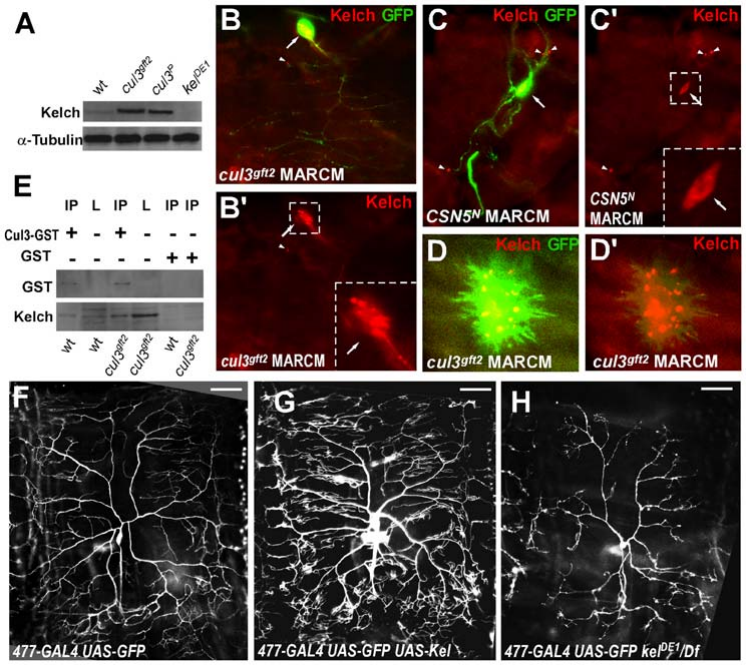
Kelch levels are critical for dendritic branching, and regulated by COP9^Cullin3^. (A) Western blot showing that Kelch is accumulated in *cul3* mutants. α-Tubulin was used as a loading control. (B, C) Kelch over-accumulates in *cul3* and *CSN5*-mutant neurons, and is virtually undetectable in the adjacent non-mutant neurons. (B′) and (C′) show Kelch (red) staining only from the panels (B) and (C). Cell bodies are marked by arrows, accumulation of Kelch at the terminal branches is pointed by arrowheads, magnified parts are marked by the dotted boxes. GFP is green, Kelch is red. (D, D′) A *cul3*-mutant epithelial cell with clumps of Kelch. (E) Kelch and Cullin3 interact physically. Complex containing the Cullin3-GST fusion protein was pulled down from wt or *cul3*-mutant larvae lysates. Presence of Kelch and GST-Cullin3 fused protein in the complex was determined by anti-Kelch and anti-GST, respectively. (F) A ddaC neuron visualized by the *477-GAL4*-driven expression of *UAS-GFP*. (G) Expression of *UAS-Kelch* under the control of *477-GAL4* stimulates dendritic branching. (H) In *kel* mutants, ddaC dendritic tree is simpler.

To identify downstream targets for Cullin3 in neurons, we tested several *Drosophila* BTB domain proteins, including the actin crosslinking BTB-domain protein Kelch. First we estimated the levels of Kelch expression in mutants for *cul3*. Because *cul3* mutants die in first and second instar larvae we used freshly hatched 1 instar *cul3* larvae from two independent alleles and the same age wild type larvae as a control. We detected that Kelch is highly accumulated in total *cul3* lysate, if compared to the wild type (Figure 3A). This indicates that BTB-domain protein Kelch can serve not only as an adaptor for Cullin3-based ligases but itself is degraded via Cullin3-based ubiquitin ligase.

To look at subcellular localization of Kelch to determine whether Kelch accumulates specifically in cells that lack *cullin3*, we immunostained third instar larvae containing *cullin3* MARCM clones with antibodies to Kelch. We detected a strong accumulation of Kelch in *cul3-*mutant neurons, while in the neighboring, non-mutant neurons, it was undetectable (Figure 3B). Similar results were detected in *CSN5-*mutant neurons: the Kelch levels were elevated in cells that lack *CSN5* and were virtually undetectable in the adjacent non-mutant (i.e. not labeled by presence of GFP) neurons (Figure 3C). In addition, in part of the tested neurons and in *cul3*-mutant epithelial cells, Kelch was aggregated in clusters (Figure 3B, D).

Specific accumulation of the actin-crosslinking protein Kelch in *cul3*-mutants identified Kelch as a target for Cullin3-mediated protein degradation in neurons, suggesting that Kelch is likely to be responsible for *cullin3* phenotype in neurons.

### Cullin3 and Kelch interact physically

To test whether Cullin3 and Kelch physically interact with each other, we performed GST pull-down experiment. We purified the Cullin3-GST fusion protein or GST control from bacteria and mixed with lysates from wild type, as well as *cul3-*mutant larvae with their abundance of Kelch. Cullin3 and associated proteins were immunoprecipitated using GST beads and analyzed on western blot. This approach revealed a Cullin3-Kelch association in vitro (Figure 3E).

### Overexpression of Kelch increases dendritic branching

Because we found that loss of cullin3 leads to over-branching along with over-accumulation of Kelch, we were interested in whether the elevated levels of Kelch are responsible for the extra branches. To test if overexpression of Kelch can mimic the cullin3-mutant phenotype, we generated a transgenic line carrying pUAST-Kelch construct that produces full-length Kelch protein.

Specific expression of the pUAST-Kelch using the ddaC neuron specific driver 477-GAL4 [49] was able to stimulate dendritic branching similar to the cullin3 phenotype. Dendritic branching was increased by 30% in the number of ends after the ectopic expression of Kelch (Figure 3G; 3F - control). More dramatic effect was achieved when larvae expressing 477-GAL4 UAS-Kelch were kept at 29°C to enhance the GAL4-driven expression.

Thus, these findings demonstrated that an excess of Kelch mimics the cullin3-mutant phenotype, thereby strengthening the idea of Kelch as a downstream target for Cullin3-mediated proteolysis in dendrites.

### Loss of kelch inhibits dendritic branching

In *Drosophila*, Kelch is known to regulate actin crosslinking during ovarian ring canal growth, and in actin-based cytoskeleton rearrangements [50], [51], but it has not been reported to be involved in dendritic branching. Our experiments suggest that Kelch is normally acting in dendrites. To further investigate the role for Kelch in neurodevelopment, we looked at the *kelch* loss of function phenotype. The *kel* null allele, *kel^DE1^*, is female sterile because of defects in actin-rich ring canals in ovary development [52], [53].

To test whether *kelch* affects dendritic branching, we first visualized all the dendritic arborization neurons with *477-GAL4 UAS-GFP* in *kel^DE1^* homozygous larvae or *kel^DE1^* over *kel* deficiency. This revealed that ddaC neurons in *kel^DE1^* mutants in third instar larvae show fewer dendritic branches (Figure 3H).

Concomitantly, we also determined that about a third, 35%, of *kel^DE1^* homozygous embryos do not survive into adult stage. This might suggest that this lethality is associated with incomplete penetrance of *kelch* phenotype. Then, severe defects in neurons could not be detected because of reduced viability of *kel^DE1^* homozygotes.

**Figure 4:**
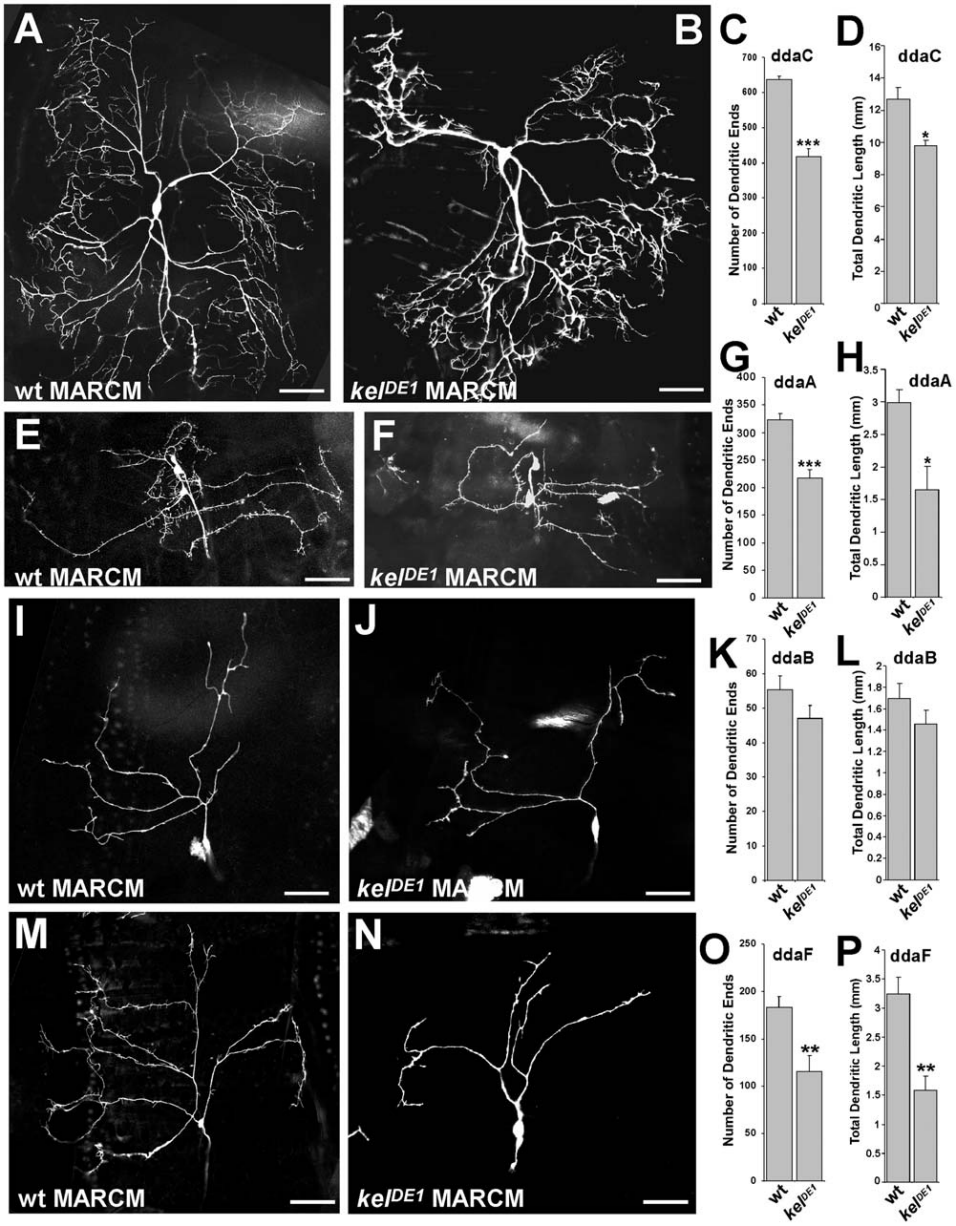
Loss of *kelch* inhibits dendritic branching. (A, E, I, M) Wild type ddaC, ddaA, ddaB, ddaF neurons, respectively. MARCM clones of *kelch^DE1^* in ddaC (B), ddaA (F), ddaB (J), ddaF (N) neurons show reduced dendritic elaboration. (C, G, K, O) Quantifications of terminal dendritic ends in wild type and *kel^DE1^* mutant ddaC, ddaA, ddaB, ddaF neurons, respectively. Scale bar: 50 µm. (D, H, L, P) Quantifications of total dendritic length in wild type and *kel^DE1^* mutant ddaC, ddaA, ddaB, ddaF neurons, respectively. ***: p<0.001, **:p<0.01, *: p<0.02.

To clarify this issue, we used the MARCM technique to generate *kel^DE1^*-mutant single-cell clones in otherwise non-mutant larvae. As expected, we found that loss of *kelch* caused a range of defects in dendritic branching (Figure 4), confirming that *kelch* mutant phenotype is not fully penetrant in neurons. In *kel^DE1^*-mutant neurons, we observed a gradient of phenotypes with different levels of abnormalities ranging from mild to severe defects. Detailed analysis of mutant phenotypes of ddaA, ddaB, ddaC, ddaF neurons showed that loss of *kel* causes reduced dendritic branching (Figure 4C, G, K, O). In particular, this phenotype represented fewer and shorter fine branches. Total dendritic length was also reduced in *kel* mutant clones (Figure 4D, H, L, P). Average total dendritic area for ddaC neurons was reduced in *kel* mutants to ∼82% when compared to wild type (p = 0.0005), (Table 1).

### Loss of *kel* suppresses *cul3* effect

We determined that Cullin3 and Kelch interact physically and, if mutated, have the opposite effect on dendritic branching. These data along with Kel over-accumulation in cul3 mutant neurons strongly suggest that dendritic branching is under the control of Cullin3-mediated proteolysis of Kelch. If this were correct, decreasing Kelch levels in *cullin3* mutants would act to rescue mutant phenotype. Thus, to test whether loss of *kelch* can shift *cullin3* phenotype in neurons toward wild type, we used MARCM technique to make single-cell clones that lack both *cullin3* and *kelch* simultaneously.

**Figure 5:**
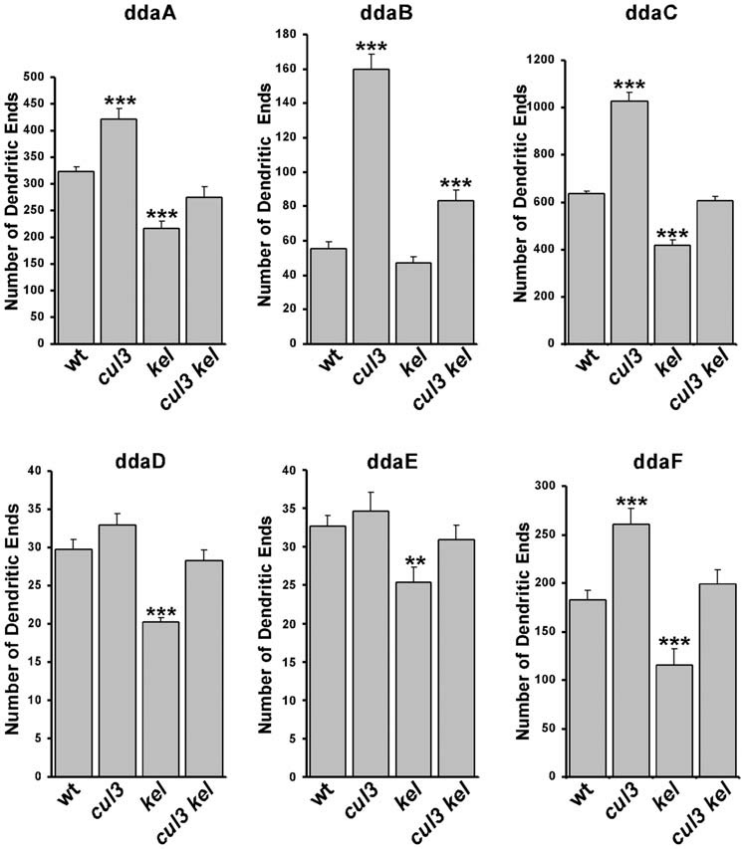
*cul3* and *kel* interact genetically, mutations in *kel* rescue the *cul3*-mutant phenotype. Quantification of dendritic ends in wild type, *cul3^gft2^*, *kel^DE1^*, and *cul3^gft2^ kel^DE1^* double-mutant clones in ddaA (A), ddaB (B), ddaC (C), ddaF (D), ddaE (E), and ddaD (F) MARCM neurons. Note: small protrusions were not counted in *cul3*-mutant ddaE and ddaD neurons. Decreasing levels of Kelch in *cul3^gft2^ kel^DE1^* double-mutant single-cell clones partially or completely rescued the *cul3*-mutant phenotype. ***: p<0.001, **: p<0.01.

As expected, quantification analysis of the number of dendritic ends in double *cullin3*-*kelch* clones in ddaB, ddaC, ddaF, ddaE, and ddaD neurons showed at least partial mutual rescue of the single *cullin3* or *kelch* phenotypes (Figure 5). The most obvious effects were observed in ddaC and ddaF neurons. Reduction of Kelch and Cullin3 simultaneously in these neurons led to nearly normal phenotype. In ddaB neurons, *cullin3 kelch* double clones also showed strong reduction of the excessive branching. In normally less-branched ddaE and ddaD neurons, the effect of reducing Kelch in *cul3* was much weaker (the small protrusions were not counted). Taken together, lower levels of Kelch supress dendritic over-branching caused by the loss of *cullin3*, suggesting that Kelch is a major target for Cullin3 in neurons.

### Loss of *cullin3* combined with overexpression of Kelch further stimulates branching

We have shown that stabilization of Kelch, either from impaired Cullin3-mediated degradation or *GAL4*-driven overexpression, leads to excessive branching. To investigate whether the increased Kelch levels in combination with impaired C*ullin3*-dependent Kelch turnover is able to stimulate additional branches, we generated double clones of *cullin3* mutants and *UAS-Kelch* expression. In these double *cullin3*-mutant/UAS-Kelch neurons, stabilized endogenous Kelch and *GAL4*-driven UAS-Kelch would act synergistically, presumably generating a stronger phenotype when compared to the individual phenotypes caused by either *cullin3* mutations or UAS-Kelch expression.

**Figure 6:**
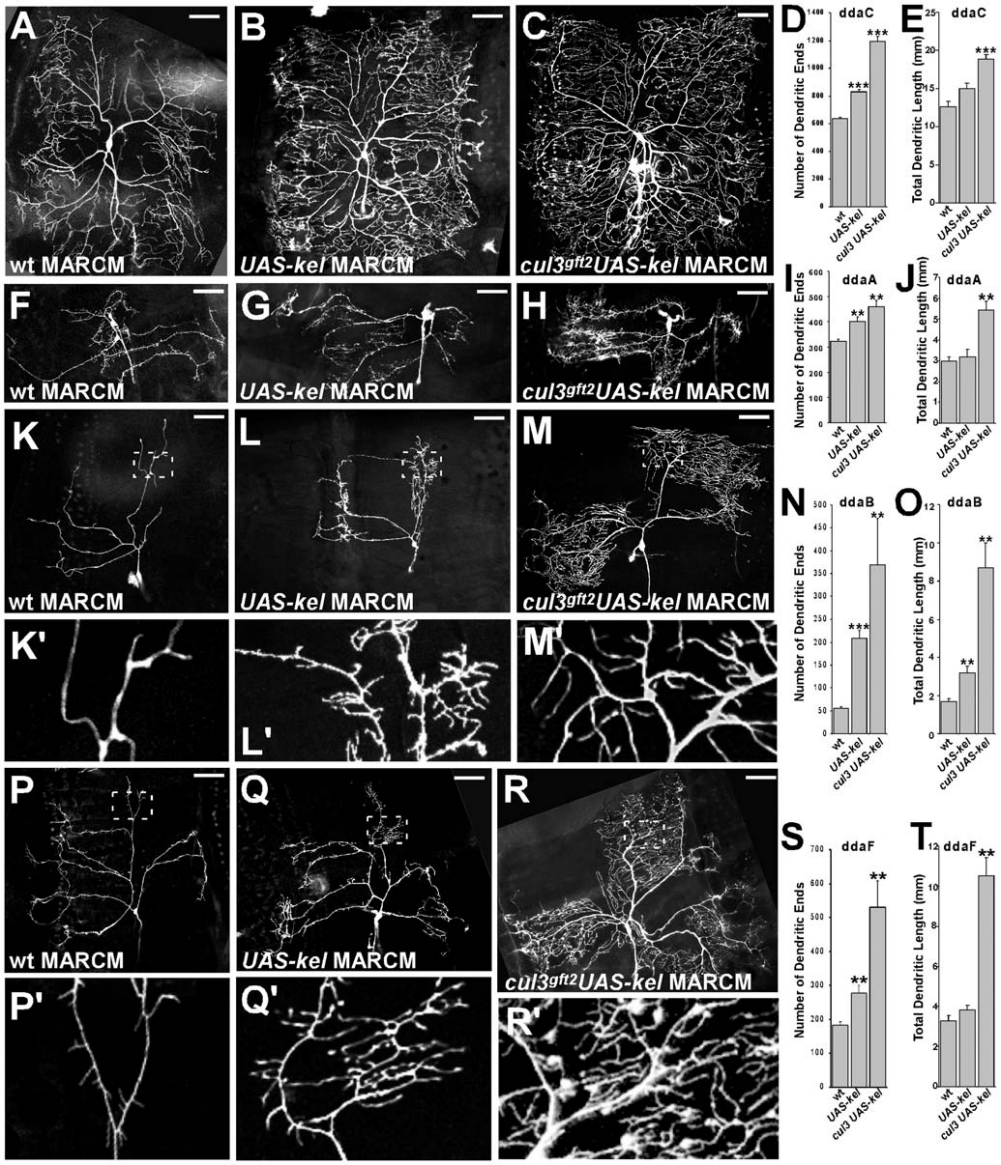
Kelch stimulates dendritic elaboration in a Cullin3-dependent manner. (A, F, K, P) MARCM clones of wild type ddaC, ddaA, ddaB, ddaF neurons, respectively. (B, G, L, Q) MARCM clones of *UAS-Kelch* in these types of neurons, Kelch overexpression stimulates dendritic branching. (C, H, M, R) Double clones of *cul3* and *UAS-Kelch* in ddaC, ddaA, ddaB, ddaF neurons. Simultaneous Kelch overexpression and loss of Cullin3 function resulted in increased branching phenotypes, especially in ddaB (M) and ddaF (R) neurons. Scale bar: 50 µm. (D, I, N, S) Quantifications of terminal dendritic ends in wild type, *UAS-Kelch*, and *cul3 UAS-Kelch* double clones in ddaC, ddaA, ddaB, ddaF neurons, respectively. (E, J, O, T) Quantifications of total dendritic length in wild type, *UAS-Kelch*, and *cul3 UAS-Kelch* double clones in ddaC, ddaA, ddaB, ddaF neurons, respectively. ***: p<0.001, **: p<0.01, *: p<0.02.

As predicted, mosaic neurons with simultaneous loss of *cullin3* and overexpression of Kelch demonstrated more dramatic phenotype. Elevation of Kelch levels in combination with lack of its downregulation led to strong over-branching and elongation defects (Figure 6). The most dramatic effect of the increased levels of Kelch was detected in ddaB and ddaF neurons. Double MARCM clones in these neurons demonstrated strong overproduction of dendritic branches accompanied by the unprecedented stimulation of the fine dendrite growth when compared to wild type, *cullin3*-mutants, or UAS-Kelch expression (Figure 6M, vs 6 K, L; 6R, vs and 6P, Q). In addition, in *cul3 UAS-Kelch* double MARCM clones we detected significant increase in both total dendritic length (Figure 6E, J, O, T) and total dendritic area (Table 1).

We used Reversed Strahler analysis [54] to analyze dendritic branching complexity. We calculated branch orders for the ddaA, ddaB and ddaF neurons (Table 2). In *cul3*-mutant clones or clones with *UAS-kel* overexpression, all these neurons demonstrated higher branching complexity when compared to control. In particular, we detected additional branch orders as well as additional branching in each order (Table 2). Combination of *cul3* mutant clones with expression of *UAS-kel* resulted in further increased dendritic arbor complexity (Table 2). For example, we observed as many as 7 branch orders in some ddaB *cul3 UAS-kel* clones vs 4 in control, and a dramatic increase in the number of branches. In contrast, loss of *kel* led to the simpler dendritic trees, fewer branches and in some cases reduction in branch order number (ddaA and ddaF neurons) (Table 2).

**Table 2:** Reversed Strahler analysis.

Neuron	Genotype	1st order	2nd order	3rd order	4th order	5th order	6th order	7th order (terminal)
**ddaA**	wt	-	-	**1**	**4.6**+/−1.5	**15.3**+/−3.5	**62.7**+/−10.2	**335**+/−62.4
	*cul3[gft2]*	-	-	**1**	**4.7**+/−0.6	**19**+/−3.6	**91**+/−14.1	**458.7**+/−91.5
	*kel[DE1]*	-	-	**0.7**+/−06	**3**+/−1	**9.3**+/−4.9	**40**+/−15.1	**213.7**+/−83.5
	*UAS-kel*	-	-	**1**	**4.3**+/−0.6	**18**+/−2.6	**87.7**+/−9.1	**431**+/−51.2
	*cul3;UAS-kel*	-	**0.3**+/−0.6	**2**+/−1.7	**5.6**+/−2.1	**19**+/−5.2	**98**+/−22.7	**480**+/−119
**ddaB**	wt	-	-	-	**1**	**3.7**+/−0.3	**10**+/−1	**51**+/−6.7
	*cul3[gft2]*	-	-	**0.7**+/−0.6	**2,7**+/−0.6	**8.3**+/−1.5	**45.3**+/−5.6	**167.7**+/−61.2
	*kel[DE1]*	-	-	-	**1**	**3.3**+/−0.6	**10.7**+/−1.5	**45.3**+/−6.7
	*UAS-kel*	-	-	**1**	**3.3**+/−0.6	**9.3**+/−2.1	**45.3**+/−9.5	**172.7**+/−43.1
	*cul3;UAS-kel*	**0.3**+/−0.6	**1.3**+/−0.6	**3.3**+/−0.6	**11.3**+/−3.2	**38.6**+/−13.8	**114.3**+/−64.7	**382.7**+/−168.4
**ddaF**	wt	-	**0.3**+/−0.6	**1.3**+/−0.6	**5**+/−1.7	**14.7**+/−4.5	**51.3**+/−14.6	**189.7**+/−39.7
	*cul3[gft2]*	-	**0.7**+/−0.6	**2.3**+/−0.6	**7**+/−1	**20.7**+/−1.5	**74.7**+/−8.1	**274**+/−54.6
	*kel[DE1]*	-	-	-	**1**	**4**+/−1	**15**+/−1	**109.3**+/−32.5
	*UAS-kel*	-	**0.3**+/−0.6	**1.7**+/−1.2	**5.6**+/−1.5	**15.7**+/−4.5	**52.7**+/−16.8	**284**+/−47.6
	*cul3;UAS-kel*	**0.3**+/−0.6	**1.6**+/−1.2	**5**+/−1	**13.3**+/−5.1	**46.7**+/−14.6	**138.7**+/−80.9	**589.7**+/−305.4

Values are the mean (± standard deviation) number of total dendritic branches in each order. The total number of neurons observed is indicated in parentheses. “–” indicates order that was not observed for the particular genotype.

**Figure 7:**
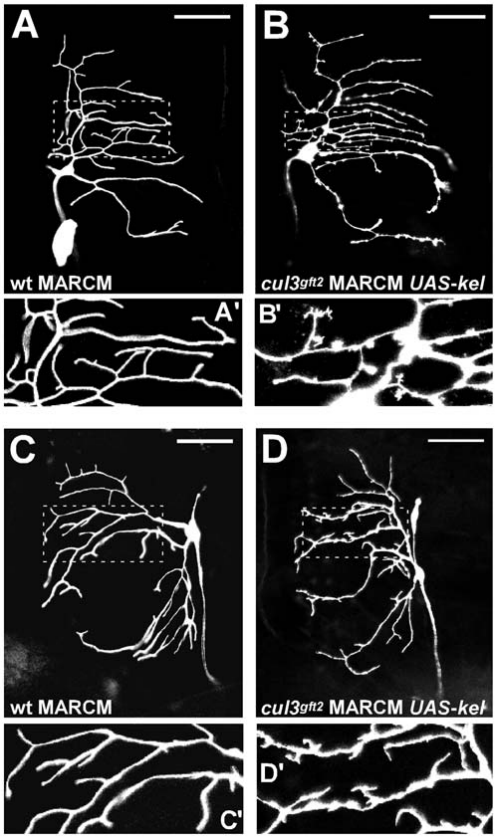
Stabilization of Kelch promotes dendritic protrusions. (A, C) Wild type ddaE and ddaD neurons. (B, D) ddaE and ddaD neurons of *cul3 UAS-Kelch* double clones. (A′), (B′), (C′) and (D′) are magnified fragments of panels (A), (B), (C) and (D). Impaired degradation of Kelch expression led to the second level protrusions in normally smooth ddaE and ddaD neurons.

In ddE and ddaD neurons, double clones also caused a remarkable phenotype. Although we did not detect dramatic enlargement of small protrusions compared to *cul3*-mutant or UAS-Kelch-expressing neurons, we found that these structures in *cullin3*/UAS-Kelch neurons generated additional levels of branching. In particular, the ectopic small protrusions sometimes produced secondary branches. Besides, general appearance of the mutant neurons was affected, as well: the diameter of the major branches was uneven (Figure 7).

Thus, we conclude that the Cullin3-mediated machinery is a chief mechanism of Kelch turnover in dendrites. It appears that if the Cullin3 function is impaired, dendritic branching directly correlates with levels of Kelch expression.

## Discussion

We found that Cullin3 acts to prevent neurons from over-branching. When the function of Cullin3 is affected, many more additional dendritic ends are formed. Our study revealed that Kelch is a downstream substrate for Cullin3-dependent degradation in *Drosophila* PNS neurons. Over-accumulation of Kelch leads to increased dendritic branching and growth, while mutations in *kelch* inhibit these processes.

### CSN^Cullin3^, dendrites and disease

It has been widely accepted that chemical and structural modifications in dendritic spines underlie much of the plastic changes in the brain in response to learning and experience [16], [55]. Growing evidence suggests that abnormal, higher or lower, number of dendritic spines contributes to mental disorders grouped as autism spectrum disorders [14], [56].

Our results identify CSN as a critical regulator of the balance of dendritic branching. We found that CSN stimulates dendritic branching via Cullin1 control of transcription (63) and prevents dendrites from over-branching and overgrowth via Cullin3 control of actin rearrangements. Because mental disorders are often accompanied by a misbalanced dendritic appearance [3], [56], the role of CSN in keeping the proper degree of dendritic arborization in the human brain could be central. Given that dendritic pattern is a fundamental determinant of neuronal wiring, it is not surprising that CSN has been found compromised in several mental retardation syndromes and neurodegenerative diseases [32], [33], [34], [35], [36], [37], [38], [39], [40], [41].

Although the neuropathology of the impaired CSN system, including Cullin3-dependent, has not been elucidated, the role of Cullin3 found in this study to prevent uncontrollable dendritic arborization might give a clue about the mechanisms of how the CSN pathway is involved in brain disorders. There are examples of excessive dendritic elaboration in patients with similar debilitating diseases. For instance, increased numbers of dendritic spines, which also appear longer and thinner than normal, were described in patients with Fragile X syndrome and were subsequently confirmed in mouse models of Fragile X syndrome [3]. Similarly, increased numbers of dendritic spines were found in brains of autistic patients with severe mental retardation [6]. Despite the fact that the particular mechanisms leading to dendritic defects could be different, their destructive effect on mental development appears to be translated via abnormal stimulation of dendritic branching.

### Actin remodeling in dendrites

The actin cytoskeleton has long been suspected to be crucial in controlling the development and stability of dendritic spines, as dendritic spines are highly enriched for actin. However, the cytoskeletal requirements of dendritic branching, growth, and guidance have not been examined as extensively as, for instance, those of axons [2].

In *Drosophila*, establishing the dendritic tree is a highly dynamic process that is also characterized by extension and retraction of branches, followed by stabilization and growth [42]. Our results suggest that Cullin3 is involved in regulating these processes. Because loss of Cullin3 leads to over-branching, Cullin3-mediated protein degradation acts as a negative regulator, or suppressor, of dendritic branching.

We determined that loss of Cullin3 in neurons leads to accumulation of Kelch. The fact that Kelch, an actin remodeling protein, is involved in these processes provides an explanation as to how this regulation occurs. Kelch is well known to bundle and stabilize actin filaments during ring canal growth in *Drosophila* ovaries [50], suggesting that Kelch regulates dendritic morphogenesis via stabilization of actin filaments in protrusions. Apparently, excess Kelch promotes new protrusions or/and stabilizes the structures that otherwise would retract. Because mutations in *kelch* demonstrated fewer branching, loss of Kelch-dependent actin filament stabilization leads to reducing new protrusions or failure to stabilize the normal amount of dendritic branches, or both.

Severe defects detected here in the length of dendritic branches in response to stabilization of Kelch indicate that Kelch is involved in dendritic growth. This effect was especially dramatic when the Kelch protein was overexpressed in *cullin3*-mutant neurons (Figure 6). Apparently, endogenous and ectopic Kelch were acting synergistically to promote actin filament stabilization, which led to the uncontrollable growth of the fine branches.

It is remarkable that *kelch* and *cullin3* are involved in development of every type of the DA neurons in *Drosophila* PNS. This might suggest that Cullin3^Kelch^ regulates actin remodeling in dendrites in a general, neuron type independent manner.

### Kelch and neurologic disease

Although our current knowledge about physiological functions of Kelch proteins is very limited, it appears that proteins containing the Kelch domain are important in mammalian neurodevelopment and have been implicated in human disorders.

The mammalian BTB-Kelch gene *gigaxonin* is mutated in giant axonal neuropathy, a severe autosomal recessive sensorimotor neuropathy affecting both the peripheral nerves and the central nervous system and is characterized by cytoskeletal neurofilament disorganization [57]. mRNA expression of the Kelch-like ECH-associated protein 1 (Keap1) was increased in the primary motor cortex in samples from amyotrophic lateral sclerosis patients, suggesting that this may contribute to chronic motor neuron degeneration [58]. Immunohistochemical studies of another Kelch-related protein, Actinfilin, in rat brain sections demonstrated that it is broadly expressed in neurons of most regions of the brain, suggesting that Actinfilin may be a key player in the actin-based neuronal function [59]. Targeted deletion of the actin-binding protein Kelch-like 1 (KLHL1) gene in Purkinje neurons resulted in dendritic deficits in these neurons, abnormal gait, and progressive loss of motor coordination in mice [60].

Taken together, evolutionarily conserved Kelch domain proteins may represent a general actin-remodeling pathological target in neurological disorders with altered cytoskeletal network. The connection found in our study among Cullin3^Kelch^ protein degradation system, actin remodeling, and dendritic density is an important step to a comprehensive understanding of the multilayer regulatory role for CSN in neuronal wiring.

## Materials and Methods

### Fly stocks and genetic crosses

Most of the fly lines were raised at 25°C using standard food medium. Some experiments were performed at 29°C as specified. The following fly stocks were used: *cul3^gft2^*; *cul3^P^ (cul3^06430^)*; *kel^DE1^; Df(2L)H20*; *109(2)80-GAL4*, *477-GAL4, UAS-mCD8::GFP*; *C155*-*GAL4*, *UAS-mCD8::GFP, hsFLP*; and *tubP-Gal80*, *40AFRT*/*CyO*.

### Generation of transgenic fly lines

The full-length *Drosophila cullin3* and *kelch* cDNA were amplified by polymerase chain reaction (PCR) using primers specific to *cul*-*3* (5′-AAAAGCGGCCGCGTTGAAAACAGCAACAAATC-3′ and 5′-AAAATCTAGATCCTGCTTAAGACGCTCCTG-3′) and *kel* (5′-AAAGCGGCCGCGATCCGTTTCGGATGATAG-3′ and 5′-TGCATATTGCTTAAAGTGGTTACGG-3′), digested with NotI and XbaI, and cloned into the pUAST vector [61]. Inserts were verified by sequencing and these plasmids were used for the generation of transgenic fly lines.

### Single-neuron MARCM analysis

Single-neuron MARCM analysis was performed as previously described [43]. Briefly, *cul3^gfp2^*, *cul3^06430^*, and *kel^DE1^* were recombined onto the chromosome containing *40AFRT*. *C155*-*GAL4*, *UAS-mCD8::GFP, hsFLP*; and *tubP-Gal80*, *40AFRT*/*CyO* were crossed with *C155*-*GAL4*, *UAS-mCD8::GFP, hsFLP*; and *cul3^gfp2^*, *40AFRT*/*CyO*. Other alleles were analyzed in similar crosses. To generate MARCM clones, embryos from these crosses were collected at 25°C for 2–3 h. They were incubated at 25°C for 3 additional hours aiming to cover the timing of mitotic events in the PNS. Then, to induce mitotic recombination using the FLP/FRT system, the embryos were heat-shocked in a 37.5°C water bath for 40 min. Developing embryos were kept at 25°C for 3–4 days, and then third instar larvae were examined for GFP-labeled clones.

### Western blot and Iimmunohistochemistry

First instar wild type and *cul3*-mutant larvae (marked by absence of balancer with GFP) were collected and used for Western immunoblotting with standard technique. Briefly, proteins were extracted in 1xLaemmli's buffer under reducing conditions, separated in 10% SDS-PAGE, transferred onto PVDF membrane (Biorad) and blocked in 5% nonfat dry milk according to manufacturer's specifications. Membranes were incubated with monoclonal antibodies to Kelch (obtained from Developmental Studies Hybridoma Bank and used at 1∶100 dilution). For immunostaining, third instar larvae containing single-neuron clones were collected, dissected, fixed in 3.7% formaldehyde in for 15 min, and permeabilized in 0.1% Triton X-100 in phosphate-buffered saline. Anti-Kelch antibodies were used at 1∶10 dilution and Cy-3 fluorescent conjugated goat anti-mouse (Jackson Laboratories, 1∶100) was used as the secondary antibody.

### In vitro binding assay

For binding assay, *Drosophila cul3* sequence was amplified by PCR using following primers: AAAAGAATTCATGAATCTGCGGGGAAATC and AAAAGCGGCCGCTGCTTAAGACGCTCCTGCT, digested with EcoRI and NotI, and cloned into pGEX-4T-1 vector. GST-cul3 construct was immobilized on Glutatione Sepharose 4B beads (Amersham Biosciences) and incubated with total wild-type or *cul3*-mutant larval lysate following manufacturer's protocol. Complexes were then sequentially resolved via SDS-PAGE and immunoblotted. Antibodies to Kelch or GST were used to detect the presence of Kelch, GST-cul3 or GST.

### Quantitative morphological analysis

The number of dendritic ends was counted manually. Total dendritic area was defined as a polygon between most distal dendritic ends and measured in the ImageJ software (NIH). NeuronStudio [62] was used to measure total dendritic length and analyze neuron branch order. Dendrites were traced semi-automatically with careful manual corrections. The Strahler method was used as described previously [54]. For some measurements neurons were traced manually. Data are presented as means±SD. All statistical analyses were performed using Student's t-test.
